# Supplementary material for: Uptake of small extracellular vesicles by recipient cells is facilitated by paracrine adhesion signaling
Source: Nat Commun. 2025 Mar 12;16:2419. doi: 10.1038/s41467-025-57617-9 (PMC11903687; doi:10.1038/s41467-025-57617-9)
Supplement: Supplementary file 2 — Description of Additional Supplementary Files [file 41467_2025_57617_MOESM2_ESM.pdf]

Description of Additional Supplementary Files

Supplementary Movie1 (the raw data for Fig. 3b)

(Left) The 3D trajectory of single sEV particle (sEV-CD63Halo7-SF650T derived from PC-3 cells) near the recipient cell (PZ-HPV-7 cell) PM. The color of the spot indicates the z-position of the sEV particle shown in the color bar. (Right) A source movie of the trajectory shown in the left was recorded at 30 Hz.

Supplementary Movie 2 (the raw data for Fig. 4d, e)

A representative PALM movie of CAV1-mEos4b (green) was merged with a movie of single sEV particles (sEVs-CD63Halo7-SF650T) (magenta) on the living PZ-HPV-7 cell PM. Single molecules of Cav1-mEos4b and single particles of sEVs were observed at 200 Hz. The superimposed movie was created according to the method shown in Fig 4b and replayed at video rate (30 Hz).

Supplementary Movie 3 (the raw data for Fig. 4d, f)

A representative PALM movie of LAMP2C-mEos4b (green) was merged with a movie of a single sEV particle (sEVs-CD63Halo7-SF650T) (magenta) on the living PZ-HPV-7 cell PM. Colocalization events are indicated by yellow arrowheads. Single molecules of LAMP2C-mEos4b and single particles of sEVs were observed at 200 Hz. The superimposed movie was created according to the method shown in Fig 4b and replayed at video rate (30 Hz).

Supplementary Movie 4 (the raw data for Fig. 6a upper)

A typical movie, showing the recruitment of single molecules of TMR-Halo7-GPI (green) to a single particle of sEV-CD63Halo7-SF650T (magenta) on the living PZ-HPV-7 cell PM. Colocalization events were indicated by the yellow arrowhead. The movie was recorded at video rate and replayed in real-time.

Supplementary Movie 5 (the raw data for Fig. 6a bottom)

A typical movie, showing single molecules of TMR-Halo7-Integrin [illegible]1 (green) and a single particle of sEV-CD81Halo7-SF650T (magenta) on the living PZ-HPV-7 cell PM. Colocalization events were indicated by the yellow arrowhead. The movie was recorded at video rate and replayed at 10 Hz

Supplementary Movie 6 (the raw data for Fig. 6c upper)

A representative PALM movie of mEos4b-talin1 (green) was merged with a movie of a single particle of sEV-CD63Halo7-SF650T (magenta) on the living PZ-HPV-7 cell PM. Single molecules of mEos4b-talin1 and a single particle of CD63Halo7-SaraFluor650T were observed at 200 Hz. The superimposed movie was created according to the method shown in Fig. 4b and replayed at a video rate (30 Hz).

Supplementary Movie 7 (the raw data for Fig. 7a upper)

The addition of PC-3-derived sEVs induced Ca^{2+} response in PZ-HPV-7 cells. The change in the intracellular Ca^{2+} concentration was monitored using the Fluo-8H indicator. sEVs were added to the cells at 2.5 minutes after the start of observation. Ionomycin was added to the cells at 20 minutes after the start of the observation. scale bar: 20 mm. The movie was recorded at 0.05 Hz and replayed at 30 Hz.
