# Supplementary material for: Uptake of small extracellular vesicles by recipient cells is facilitated by paracrine adhesion signaling
Source: Nat Commun. 2025 Mar 12;16:2419. doi: 10.1038/s41467-025-57617-9 (PMC11903687; doi:10.1038/s41467-025-57617-9)
Supplement: Supplementary file 11 — Reporting Summary [file 41467_2025_57617_MOESM11_ESM.pdf]

nature portfolio

Corresponding author(s): Kenichi G. N. Suzuki

Last updated by author(s): Dec 20, 2024

Reporting Summary

Nature Portfolio wishes to improve the reproducibility of the work that we publish. This form provides structure for consistency and transparency in reporting. For further information on Nature Portfolio policies, see our Editorial Policies and the Editorial Policy Checklist.

Statistics

For all statistical analyses, confirm that the following items are present in the figure legend, table legend, main text, or Methods section.

n/a	Confirmed	
☐	☒	The exact sample size (*n*) for each experimental group/condition, given as a discrete number and unit of measurement
☐	☒	A statement on whether measurements were taken from distinct samples or whether the same sample was measured repeatedly
☐	☒	The statistical test(s) used AND whether they are one- or two-sided<br>*Only common tests should be described solely by name; describe more complex techniques in the Methods section.*
☐	☒	A description of all covariates tested
☐	☒	A description of any assumptions or corrections, such as tests of normality and adjustment for multiple comparisons
☐	☒	A full description of the statistical parameters including central tendency (e.g. means) or other basic estimates (e.g. regression coefficient) AND variation (e.g. standard deviation) or associated estimates of uncertainty (e.g. confidence intervals)
☐	☒	For null hypothesis testing, the test statistic (e.g. *F*, *t*, *r*) with confidence intervals, effect sizes, degrees of freedom and *P* value noted<br>*Give P values as exact values whenever suitable.*
☒	☐	For Bayesian analysis, information on the choice of priors and Markov chain Monte Carlo settings
☐	☒	For hierarchical and complex designs, identification of the appropriate level for tests and full reporting of outcomes
☐	☒	Estimates of effect sizes (e.g. Cohen's *d*, Pearson's *r*), indicating how they were calculated

Our web collection on statistics for biologists contains articles on many of the points above.

Software and code

Policy information about availability of computer code

Data collection	Western blot data: FUSION SOLO 7S.<br>Confocal microscopy:FV-1000D(Olympus).<br>TIRF microscopy:Home-built single-molecule imaging station built on Olympus IX-83 (Olympus) and NIkon Ti-E.<br>Single fluorescent-molecule tracking: WinTrack, WinATR, and WinSAT, produced in house (Komura et al., Nat. Chem. Biol. 2016; Kinoshita et al., J. Cell Biol. 2017; Morise et al., Nat. Commun. 2019)<br>Acquisitions of super-resolution microscopic images: ThunderSTORM plugin of Fiji (ver. 2.1.0/1.54f)
Data analysis	Western blot images:<br>Data were analysed by Fiji (ver. 2.1.0/1.53c).<br>Confocal microscopy images:<br>Data were analysed by Fiji (ver. 2.1.0/1.53c)<br>Analysis of super-resolution microscopic images:<br>All the software used is described in the Methods section. Data and code availability statement is now placed as an independent Subsection with the heading "Data and materials availability" .<br>Statistical analysis; Origin Pro 2018b (OriginLab)

For manuscripts utilizing custom algorithms or software that are central to the research but not yet described in published literature, software must be made available to editors and reviewers. We strongly encourage code deposition in a community repository (e.g. GitHub). See the Nature Portfolio guidelines for submitting code & software for further information.

Data

Policy information about availability of data

All manuscripts must include a data availability statement. This statement should provide the following information, where applicable:

- Accession codes, unique identifiers, or web links for publicly available datasets
- A description of any restrictions on data availability
- For clinical datasets or third party data, please ensure that the statement adheres to our policy

A full data availability statements is included in the manuscript

Research involving human participants, their data, or biological material

Policy information about studies with human participants or human data. See also policy information about sex, gender (identity/presentation), and sexual orientation and race, ethnicity and racism.

Reporting on sex and gender	N.A.
Reporting on race, ethnicity, or other socially relevant groupings	N.A.
Population characteristics	N.A.
Recruitment	N.A.
Ethics oversight	N.A.

Note that full information on the approval of the study protocol must also be provided in the manuscript.

Field-specific reporting

Please select the one below that is the best fit for your research. If you are not sure, read the appropriate sections before making your selection.

☒ Life sciences ☐ Behavioural & social sciences ☐ Ecological, evolutionary & environmental sciences

For a reference copy of the document with all sections, see nature.com/documents/nr-reporting-summary-flat.pdf

Life sciences study design

All studies must disclose on these points even when the disclosure is negative.

Sample size	We performed pilot experiments using smaller sample numbers, and after performing Student t-test, we determined the expected minimal sample size to prove or disprove the hypothesis. We generally did more experiments than this minimal number of experiments.
Data exclusions	No data exclusions.
Replication	All experimental findings were reliably reproduced.
Randomization	All sample allocations were random.
Blinding	The sample preparation and observation were mostly preformed by the same operator. Therefore, no blinding was performed.

Reporting for specific materials, systems and methods

We require information from authors about some types of materials, experimental systems and methods used in many studies. Here, indicate whether each material, system or method listed is relevant to your study. If you are not sure if a list item applies to your research, read the appropriate section before selecting a response.

Materials & experimental systems

n/a	Involved in the study
☐	☒ Antibodies
☐	☒ Eukaryotic cell lines
☒	☐ Palaeontology and archaeology
☒	☐ Animals and other organisms
☒	☐ Clinical data
☐	☐ Dual use research of concern
☒	☐ Plants

Methods

n/a	Involved in the study
☒	☐ ChIP-seq
☐	☒ Flow cytometry
☒	☐ MRI-based neuroimaging

Antibodies

Antibodies used: anti-CD63 (Cosmo Bio, SHI-EXO-M02), anti-CD81 (Santa Cruz, sc-166029), anti-CD9 (Abcam, ab263019), anti-GFP (Sigma, SAB4301138), anti-HaloTag (Promega, G9281), anti-β-actin (Thermo Fisher Scientific, MA1-140), HRP-conjugated goat anti-mouse IgG (Millipore, 12-349), HRP-conjugated goat anti-rabbit IgG (Sigma–Aldrich, A0545), HRP-conjugated donkey anti-rabbit IgG (Cytiva, NA934), anti-FAK (Abcam, ab40794), anti-Phospho-FAK(Tyr861) (Invitrogen, 44-626G), anti-CD29 (BD, 610467), anti-active form of integrin beta 1 (HUTS-4, Millipore), anti-Talin (GeneTEX, 97H6), Alexa488 conjugated anti-mouse-IgG (abcam, ab150077), Rhodamine conjugated anti-rabbit IgG (55666, Cappel), anti-Caveolin-1 (BD Transduction Laboratories, Clone 2297), anti-Galectin3(eBioscience, eBioM3/38), Alexa Fluor 488 conjugated secondary antibody against rat IgG (Thermo Fisher Scientific, A21208)

Validation: Validation was based on the data sheet of manufacturers and researchers. If necessary, additional validation was performed largely by Western blot and/or immunofluorescence.

Eukaryotic cell lines

Policy information about cell lines and Sex and Gender in Research

Cell line source(s): Human prostate cancer (PC-3; RL-1435), Human fibroblast (WI-38; CL-75), and Human prostate (PZ-HPV-7; RL-2221) cells obtained from ATCC. Human mesenchymal stem cells (MSCs; PT-2501; Lot 23TL142784) obtained from Lonza. MDA-MB-231 human breast cancer organotropic line 4175-LuT cells (kindly provided by Dr. Joan Massagué (Memorial Sloan Kettering Cancer Center (MSKCC), New York, NY, USA)

Authentication: N.A.

Mycoplasma contamination: Confirm that all cell lines were tested negative for mycoplasma contaminations

Commonly misidentified lines (See ICLAC register): N.A.

Plants

Seed stocks: N.A.

Novel plant genotypes: N.A.

Authentication: N.A.

Flow Cytometry

Plots

Confirm that:

☒ The axis labels state the marker and fluorochrome used (e.g. CD4-FITC).

☒ The axis scales are clearly visible. Include numbers along axes only for bottom left plot of group (a 'group' is an analysis of identical markers).

☒ All plots are contour plots with outliers or pseudocolor plots.

☒ A numerical value for number of cells or percentage (with statistics) is provided.

Methodology

Sample preparation	To quantify mGFP and Halo expression, cells were stained with 100 nM TMR-labeled Halo ligand for 30 minutes. After washing with culture medium and PBS, cells were harvested using cell scrapers and centrifuged at 1,400 g for 3 minutes.
Instrument	FACS Melody (BD Biosciences)
Software	FlowJo (BD Biosciences)
Cell population abundance	The purity of fluorescently labeled cell within post-sort fraction (> 95%) was determined analyzing the sorted cells in a cytometer instrument just after their sorting.
Gating strategy	1.) SSC vs. FSC gating to exclude debris. 2.) FSC-H vs. FSC-A gating to exclude doublets.<br>3.) EGFP vs. TMR gating to quantify EGFP+ and Halo7+ cells.<br>Boundaries for Gate 3 were based on a intact cell control.

☐ Tick this box to confirm that a figure exemplifying the gating strategy is provided in the Supplementary Information.
